# General anesthesia might be associated with early periprosthetic joint infection: an observational study of 3,909 arthroplasties

**DOI:** 10.1080/17453674.2019.1644069

**Published:** 2019-07-24

**Authors:** Ruben Scholten, Borg Leijtens, Gerjon Hannink, Ed T Kamphuis, Matthijs P Somford, Job L C van Susante

**Affiliations:** a Rijnstate Ziekenhuis, Department of Orthopedics, Arnhem;; b Radboud University Medical Center, Department of Operating Rooms, Nijmegen;; c Rijnstate Ziekenhuis, Department of Anesthesiology, Arnhem, the Netherlands

## Abstract

Background and purpose — Periprosthetic joint infection (PJI) remains a devastating complication following total knee or total hip arthroplasty (TKA/THA). Nowadays, many studies focus on preventive strategies regarding PJI; however, the potential role of anesthesia in the development of PJI remains unclear.

Patients and methods — All consecutive patients undergoing elective primary unilateral TKA or THA from January 2014 through December 2017 were included. Exclusion criteria included femoral fractures as the indication for surgery and previously performed osteosynthesis or hardware removal on the affected joint. Age, sex, BMI, ASA classification, type of arthroplasty surgery, type of anesthesia, duration of surgery, smoking status, and intraoperative hypothermia were recorded. Propensity score-matched univariable logistic regression analysis was used to control for allocation bias.

Results — 3,909 procedures consisting of 54% THAs and 46% TKAs were available for analysis. 42% arthroplasties were performed under general anesthesia and 58% under spinal anesthesia. Early PJIs were observed in 1.7% of the general anesthesia group and in 0.8% in the spinal anesthesia group. The multivariable logistic regression model demonstrated an odds ratio for PJI of 2.0 (95% CI 1.0–3.7) after general anesthesia relative to the propensity score-matched patients who received spinal anesthesia.

Interpretation — These results suggest a potential association between general anesthesia and early PJI. Future research using large-scale data is required to further elucidate this clinically relevant association.

Periprosthetic joint infection (PJI) is responsible for up to 25% of failed TKA and 15% of failed THA (Bozic et al. 2009, 2010). Despite the increasing awareness of certain patient characteristics that influence the risk of PJI (Kunutsor et al. 2016), the role of procedure-related factors, such as the type of anesthesia, remains to be elucidated (Berbari et al. 2017). Remarkably, the notion that anesthesia may influence the immune response was suggested as early as 1903 (Moudgil 1986). In the late 1970s and ’80s several reviews identified the ability of anesthetic agents to influence a wide variety of specific and non-specific host defenses (Moudgil 1986). However, the clinical relevance and the exact role of anesthesia in the pathogenesis of postoperative infections remains unclear (Moudgil 1986, Cruz et al. 2017).

Several studies have suggested spinal anesthesia to reduce the risk for surgical site infection (SSI) when compared with general anesthesia in THA and TKA (Liu et al. 2013, Pugely et al. 2013); however, other studies found no clear difference between the 2 types of anesthesia and their influence on SSI (Basques et al. 2015, Helwani et al. 2015, Kopp et al. 2015). Nevertheless, a recent systematic review suggested that regional anesthesia seems to decrease the risk of SSI when compared with general anesthesia (Zorrilla-Vaca et al. 2016). Despite several clues pointing to general anesthesia predisposing to infection, no studies assessing the role of anesthesia during THA and TKA with well-defined definitions of PJI have been performed.

Therefore, we investigated the relationship between type of anesthesia (i.e., spinal or general) and PJI following THA or TKA in a large-scale observational cohort study.  

## Patients and methods

All consecutive patients undergoing elective primary unilateral TKA or THA for osteoarthritis in a single general teaching hospital from January 2014 through December 2017 were retrieved from the hospital’s prospective database. Subsequent exclusion criteria were proximal femoral fracture or acetabular fracture as the indication for primary surgery and concomitant or previous hardware removal on the affected joint. Data were recorded regarding the patient’s age, sex, ASA classification, BMI, smoking behavior, type of arthroplasty surgery, type of anesthesia, surgery duration, intraoperative hypothermia, and length of stay.

Over the course of the study period a similar surgical technique was used, and no changes to the surgery protocol were implemented. Patients received prophylactic administration of 2 grams cefazolin 15 to 60 minutes prior to skin incision or tourniquet inflation, followed by 3 administrations of 1 gram after surgery with an 8-hour interval. All THAs were performed by, or under direct supervision of, 1 of 7 hip surgeons. Accordingly, all TKAs were performed by 1 of 4 knee surgeons. Several residents or trainees participated in most surgeries. All TKA patients underwent surgery while using a tourniquet, which was inflated 15 to 60 minutes after infusion of the prophylactic cefazolin and deflated after applying a pressure bandage over the affected knee. Only patients with primary implant models and no revision models were included. All TKAs were cemented and performed using a medial parapatellar arthrotomy. THA was performed using a posterolateral approach. Both cemented and uncemented THA were performed with a patient age cut-off point below 75 years for uncemented THA. The bone cement (Palacos R + G; Heraeus Medical, Hanau, Germany) used in both TKA and THA contained 0.75 grams of gentamicin per 61.2 grams of powder.

The decision to apply either general or spinal anesthesia during the arthroplasty was at the discretion of 1 of the senior anesthesiologists and based on the patient’s personal preference. Patients were extensively informed about both general and spinal anesthesia, after which they could indicate their preference. To correct for potential allocation bias introduced through this selection procedure, propensity score-based matching of cases was performed (please refer to Statistics section for further information).

Surgical duration was defined as the time between skin incision and closure. The core temperature was measured at the tympanic membrane in the operating room directly after wound closure.

Prior to discharge patients were closely monitored for signs of potential postoperative infection. Following discharge, all patients were subjected to protocolized surveillance of infection in the outpatient clinic for at least 3 months after surgery. In case of prolonged wound drainage (> 10 days), suspected (superficial) surgical site infection (SSI), or superficial wound breakdown, surgical debridement, with antibiotics and implant retention (DAIR), was performed. During DAIR, 6 periprosthetic tissue biopsies were always obtained and subsequently cultured. Superficial SSI was defined according to the Infectious Centers of Disease Control (CDC) guidelines with the presence of: (1) purulent incisional drainage, (2) positive culture of aseptically obtained fluid or tissue from the superficial wound, (3) local signs and symptoms of pain or tenderness, swelling, and erythema after the incision is opened by the surgeon (unless culture negative), or (4) diagnosis of SSI by the attending surgeon or physician based on their experience and expert opinion (Horan et al. 1992).

Until final cultures results were obtained up to 10 days after DAIR, patients were treated with intravenous antibiotics (flucloxacillin, 6g/day via continuous intravenous infusion).

PJI was diagnosed according to the major Musculoskeletal Infection Society (MSIS) criteria by means of 2 or more tissue cultures demonstrating growth of an identical pathogen (Parvizi and Gehrke 2014). If PJI was diagnosed, antibiotic therapy was adjusted accordingly in consultation with the attending microbiologist.

The primary outcome of this study was the incidence of PJI within 3 months of surgery.

### Statistics

Multiple imputation by chained equations procedures was used for missing values to increase precision and to avoid bias (van Buuren 2018). We generated 25 independent imputed datasets, as current guidance recommends that 1 imputation should be performed per percent of incomplete observations (White et al. 2011). Smoking behavior and hypothermia had 4.1% and 24% missing values, respectively, whereas other variables had less than 0.1% missing values (Table 1).

**Table 1.** Distribution of patient characteristics and missing data among the general anesthesia and spinal anesthesia groups. Values are frequency (%) unless otherwise stated

Factor	Spinal anesthesia (n = 2,279)	Missing data (%)	General anesthesia (n = 1,630)	Missing data (%)	Cumulative missing data (%)
Age, mean (SD)	70 (9.5)	0	67 (10)	0	0
Male sex	789 (35)	0	597 (37)	0	0
BMI, mean (SD)	28.7 (4.7)	0	29.7 (5.2)	2 (0.1)	2 (0.0)
ASA 1	348 (15)	0	221 (14)	1 (0.1)	1 (0.0)
ASA 2	1,614 (71)	0	1,049 (64)	1 (0.1)	1 (0.0)
ASA 3	306 (13)	0	344 (21)	1 (0.1)	1 (0.0)
ASA 4	11 (0.5)	0	15 (0.9)	1 (0.1)	1 (0.0)
Active smoker	231 (11)	101 (4.4)	223 (14)	61 (3.7)	162 (4.1)
TKA	1,082 (48)	0	716 (44)	0	0
2014	674 (30)	0	286 (18)	0	0
2015	591 (26)	0	391 (24)	0	0
2016	488 (21)	0	518 (32)	0	0
2017	526 (23)	0	435 (27)	0	0
Mean surgery duration, min (SD)	59 (16)	0	62 (16)	0	0
Hypothermia	87 (5.6)	724 (32)	54 (3.8)	223 (14)	947 (24)
PJI	19 (0.8)	0	28 (1.7)	0	0

Percentages are displayed as valid (calculated through discarding missing data) percentages.

BMI: body mass index,

TKA: total knee arthroplasty,

PJI: periprosthetic joint infection.

A difference for the risk for early PJI between cases that received spinal and those that received general anesthesia might be biased by confounding. A particularly important type of confounding in this case is “confounding by indication,” which occurs when the clinical indication for selecting a particular intervention also affects the outcome. For example, patients with more severe comorbidities (e.g., CVD) are more likely to receive general anesthesia, but they are also more likely to develop early PJI. Another type of confounding is “confounding by association”, which occurs when both exposure (i.e. type of anesthesia) and outcome (i.e. early PJI) are associated with a third variable. For example, BMI is associated both with type of anesthesia and with increasing risk of early PJI. In order to adjust for potential confounding baseline characteristics, we matched patients based on their propensity scores (Rubin 1997). The propensity score was defined as the probability of receiving general anesthesia during TJA dependent on a case’s recorded baseline characteristics. Propensity scores were estimated independently for each imputed dataset, using a logistic regression model with type of anesthesia as the dependent variable in relation to the following baseline characteristics: age, sex, BMI, ASA classification, smoking status, THA or TKA surgery, and year of surgery. The selection of which variables to include in our analyses in order to minimize bias was done using directed acyclic graphs based on the approaches described by Shrier and Pearl (Shrier and Platt 2008, Pearl 2010). A 1:1 optimal matching algorithm was applied without replacement to match exposed and non-exposed cases on their corresponding propensity scores within a caliper of 0.2 standard deviation of the logit of the propensity score (Austin 2011). A 1:1 matching on propensity score was used as it has been shown that it tends to minimize bias compared with many-to-1 matching on propensity score (Austin 2010). The balance between the two groups after matching was checked graphically and descriptively. A standardized difference of less than 10% indicates an appropriate balance (Austin 2011). Standardized differences (difference in means divided by the pooled standard deviation) of the baseline characteristics for a randomly selected matched dataset are provided in Table 2 (see Supplementary data).

On each of the 25 imputed and propensity score-matched datasets, a univariable logistic regression analysis with PJI within 3 months after surgery as the dependent variable and type of anesthesia as independent variable was performed. The resulting estimates were pooled using Rubin’s rule (Rubin 1997). Statistical analyses were performed using R 3.5.2 (R Foundation for Statistical Computing, Vienna, Austria).

**Figure:**
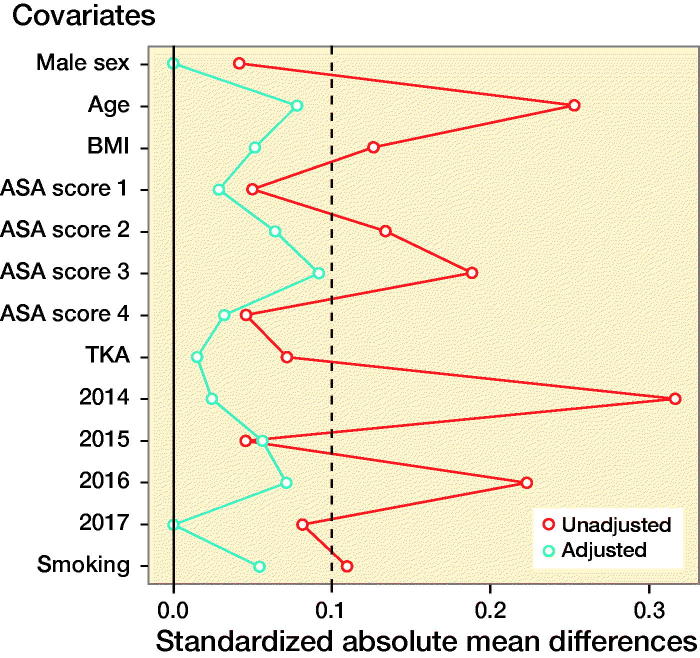
Covariate balance before (unadjusted) and after (adjusted) propensity score matching. Standardized differences less than 10% (dashed line) indicate an appropriate balance (Austin 2010).

### Ethics, funding, and potential conflicts of interest

The local institutional review board approved this study (study number: 2018-1276). No funding was received. No conflicts of interest were declared.

## Results

Between January 1, 2014 and December 31, 2017, 4,026 primary unilateral total hip and knee arthroplasties were performed. 58 THAs and 59 TKAs were excluded due to previous or concomitant hardware removal, leaving 3,909 joints consisting of 2,111 (54%) hips and 1,798 (46%) knees available for analysis.

Among all eligible patients, 1,630 (42%) arthroplasties were performed under general anesthesia and 2,279 (58%) arthroplasties were performed under spinal anesthesia. Apart from the DAIR procedures, 17 cases underwent revision surgery within 3 months of primary TJA (Table 3, see Supplementary data). None of these cases were eventually diagnosed with early PJI.

47 early PJIs were diagnosed through 2 or more positive intraoperative tissue cultures, obtained during DAIR, demonstrating an identical pathogen. 28 (1.7%) PJIs occurred in the general anesthesia group and 19 (0.8%) in the spinal anesthesia group.

The covariate balance before and after propensity score-based matching is displayed in the Figure and Table 2 (see Supplementary data). In the 1,630 patients who received general anesthesia 28 (1.7%) PJIs occurred, while in the 1,630 matched participants who received spinal anesthesia, 13–15 (0.8–0.9%) PJIs occurred, depending on imputation set.

The odds ratio for early PJI was estimated to be 2.0 (95% CI 1.0–3.7) for patients who received general anesthesia compared with matched patients who received spinal anesthesia.

Although no longer statistically significant, subsequent subgroup analysis addressing THA and TKA separately showed similar odds ratios (THA 2.1 [CI 0.99–4.6] and TKA 2.0 [CI 0.53–7.9]).  

## Discussion

Over the past decade, several studies have suggested that spinal anesthesia decreases the risk for SSI after TJA when compared with general anesthesia (Liu et al. 2013, Pugely et al. 2013). However, this remains debated since many conflicting results have been reported and there is a paucity of high-quality studies using objective criteria for SSI (Basques et al. 2015, Helwani et al. 2015, Kopp et al. 2015). The distinction between (superficial) SSI and early PJI in orthopedic surgery is far from straightforward yet clinically important. In 1999, the Centers for Disease Control (CDC) formulated definitions for superficial, deep incisional, and organ/space SSI (Mangram et al. 1999). However, there are no procedures or tests to reliably allow differentiation between these subtypes of SSI (Amanatullah et al. 2019). Furthermore, diagnostic criteria for superficial SSI such as tenderness, redness, localized swelling, and local heat are subject to interobserver variability (Allami et al. 2005). Therefore, previous studies addressing the effect of anesthesia on SSI yield less reliable results compared with this study using objectified PJI as the primary outcome measure.

The IDSA guidelines dictate vigorous surgical treatment for (suspected) SSI following TJA including surgical debridement and rinsing of the implant (Osmon et al. 2013). In previous studies these guidelines were not applied and as such the diagnosis of actual early PJI was not reliably established.

To our knowledge this is the first study using the IDSA guidelines where an association between the type of anesthesia and the incidence of objectified early PJI (using the major MSIS criteria through the availability of at least 6 periprosthetic tissue cultures in every case with suspected infection) is shown. Our results indicate an increased risk of early PJI following TJA under general anesthesia, illustrated by an odds ratio of 2 (CI 1.0–3.7). Although no longer statistically significant, subgroup analysis for the type of arthroplasty (THA or TKA) demonstrated similar confidence intervals, which indicate these results are robust and do not seem to depend on type of arthroplasty.

So far, the mechanism by which general anesthesia might increase, or spinal anesthesia might reduce the incidence of infection is not fully understood. However, increased tissue oxygenation (through reduced postoperative pain and the direct vasodilatory effect of spinal anesthesia) has been suggested as a potential mechanism in the past (Sessler 2010).

Alongside these beneficial effects on tissue oxygenation, neuraxial anesthesia is also associated with reduced blood loss, a reduced requirement for blood transfusions, and a reduced incidence of hyperglycemia. All these factors are known for their immunosuppressive effects (Guay 2006, Gottschalk et al. 2014).

Besides the suggestion of protective effects of spinal anesthesia, several aesthetic agents that are commonly used in general anesthesia may significantly inhibit leukocyte chemotactic migration, phagocytosis, lymphocyte function, inflammation, or even directly support bacterial growth in case of contamination (Moudgil 1986). Furthermore, studies comparing general and spinal anesthesia showed that in spinal anesthesia these immunosuppressive effects were minimal (Whelan and Morris 1982).

On the other hand, one could speculate regarding a potential negative effect of spinal anesthesia on the incidence of early PJI induced by intraoperative hypothermia, which has been associated with an increased incidence of SSIs in other surgical specialties and is more prevalent during spinal anesthesia (Scholten et al. 2018). However, despite the latter, spinal anesthesia was still associated with a reduced risk of early PJI in our study.

### Limitations

First, due to the observational nature of the study, confounding (by indication) cannot be precluded. To control for this potential confounding, we matched patients based on propensity scores. Although matching of patients was successfully performed based on a subset of baseline characteristics, differences could theoretically still exist in unmeasured covariates (e.g., diabetes mellitus, rheumatoid arthritis, and anticoagulant usage in this study) resulting in residual confounding. Anticoagulant therapy, for example, is generally considered to be a contra-indication for the application of spinal anesthesia. This may have caused allocation of anticoagulant users to the general anesthesia group. However, since protocols for perioperative interruption of anticoagulant use (with or without bridging) are readily available and mandatory regarding elective TJA in our clinic, anticoagulant therapy is unlikely to cause allocation of patients towards the general anesthesia group. Furthermore, both diabetes and rheumatoid arthritis do not influence the choice for either spinal or general anesthesia in our hospital.

Another limitation is the fact that our data are sourced from 1 hospital only. Therefore, the major question remains whether our data and the conclusions drawn will be reproducible in studies on, for example, national joint registries. However, on the other hand this last limitation warranted that complete follow-up could be guaranteed and that no PJIs could have been missed.

In conclusion, this is the first study to suggest a potential association between general anesthesia and an increased risk of early PJI. This clinically relevant finding should encourage the setting up of future research using (multi-center) randomized large-scale data and national joint registry studies.

### Supplementary data

Tables 2 and 3 are available as supplementary data in the online version of this article, http://dx.doi.org/10.1080/17453674.2019.1644069

## Supplementary Material

Supplemental Material
