# Supplementary material for: General anesthesia might be associated with early periprosthetic joint infection: an observational study of 3,909 arthroplasties
Source: Acta Orthop. 2019 Jul 24;90(6):554–8. doi: 10.1080/17453674.2019.1644069 (PMC6844397; doi:10.1080/17453674.2019.1644069)
Supplement: Supplemental Material [file IORT_A_1644069_SM3091.pdf]

# Supplementary data

**Table 2. Distribution of number and corresponding proportions or means and corresponding standard deviation of patient characteristics and comorbidities among the general anesthesia and spinal anesthesia groups before and after matching based on propensity scores for a randomly selected imputation set. Values are frequency (%) unless otherwise stated**

| | Before matching | | | After matching | | |
|---|---|---|---|---|---|---|
| Factor | Spinal anesthesia (n = 2,279) | General anesthesia (n = 1,630) | Standardized difference | Spinal anesthesia (n = 1,630) | General anesthesia (n = 1,630) | Standardized difference |
| Age, mean (SD) | 70 (9.5) | 67 (10.1) | –0.25 | 68 (9.7) | 67 (10.1) | –0.08 |
| Male sex | 789 (35) | 597 (36.6) | 0.04 | 607 (37 ) | 597 (37) | 0.00 |
| BMI, mean (SD) | 28.71 (4.7) | 29.7 (5.2) | 0.13 | 29.14 (4.9) | 29.37 (5.2) | 0.05 |
| ASA 1 | 348 (15) | 221 (14) | –0.05 | 236 (15) | 221 (14) | –0.03 |
| ASA 2 | 1,614 (71) | 1,049 (64) | –0.14 | 1,105 (68) | 1,049 (64) | –0.06 |
| ASA 3 | 306 (13) | 344 (21) | 0.19 | 279 (17.1) | 344 (21.1) | 0.09 |
| ASA 4 | 11 (0.5) | 15 (0.9) | 0.05 | 10 (0.6) | 15 (0.9) | 0.03 |
| Active smoker | 231 (11) | 223 (14) | 0.11 | 201 (12) | 232 (14.2) | 0.05 |
| TKA | 1,082 (47.5) | 716 (43.9) | –0.07 | 735 (45.1) | 716 (44) | –0.02 |
| 2014 | 674 (30) | 286 (18) | –0.32 | 298 (18) | 286 (18) | –0.02 |
| 2015 | 591 (26) | 391 (24) | –0.05 | 425 (26) | 391 (24) | –0.06 |
| 2016 | 488 (21) | 518 (32) | 0.22 | 465 (29) | 518 (32) | 0.07 |
| 2017 | 526 (23) | 435 (27) | 0.08 | 442 (27) | 435 (27) | 0.00 |

BMI: body mass index, TKA: total knee arthroplasty.

**Table 3. Number of patients requiring revision surgery within 3 months of index surgery**

| Cause | n |
|---|---|
| Recurrent dislocation | 7 |
| Periprosthetic fracture | 8 |
| Spinout of insert | 1 |
| Femoral stem subsidence | 1 |
| Total | 17 |
